# JACMP 2005–2009: Growth, quality, and opportunity

**DOI:** 10.1002/acm2.14445

**Published:** 2024-06-18

**Authors:** Timothy D. Solberg

**Affiliations:** ^1^ Department of Radiation Oncology University of Washington Seattle Washington USA

With the formation of the journal in 2000, the first 5 years were focused on enhancing the financial profile by transitioning to a new publisher, Multimed, the adoption of the Public Knowledge Project Open Journal Systems (OJS) manuscript management software, and generating revenue in the form of web site banner advertisements. Challenges behind us, in the subsequent 5 years, 2005−2009, we were able to focus on quality of content, growth, and new opportunities. While the JACMP continued to publish only four issues per year, the number of manuscripts and page numbers doubled over the 5 year period (Figure 1). Likewise, the number of manuscripts with 15 citations or more citations (current as of 20/5/2024) grew from 8 in 2005 to 26 in 2009. The most highly cited manuscript in each issue is given in Table 1.

**FIGURE 1:**
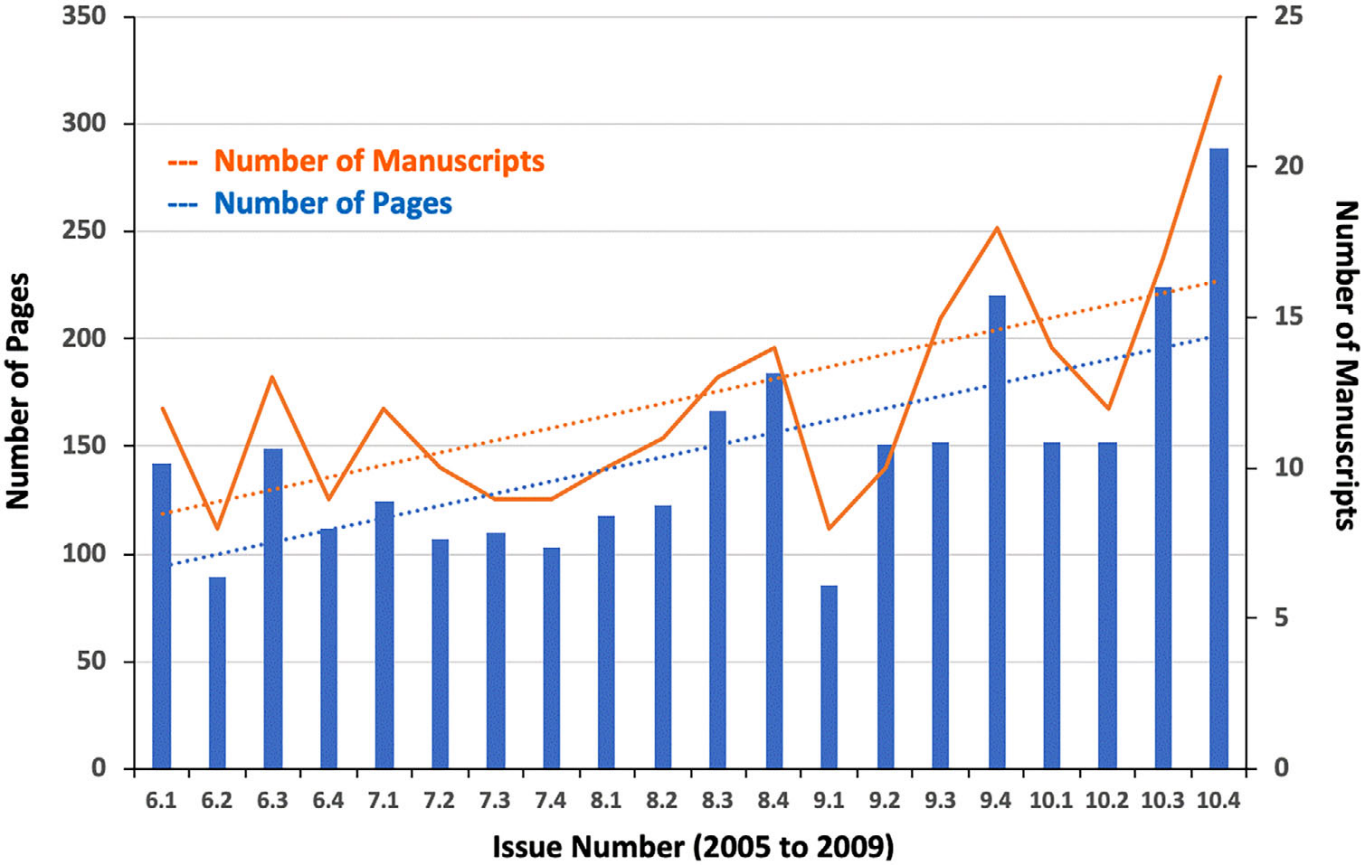
Number of manuscripts and pages published in each issue from 2005 to 2009. Dashed lines show the linear trend. The notable decrease in publications in issue 9.1 reflects the transition of editor‐in‐chief at that time.

**TABLE 1:** The most highly cited JACMP manuscript in each issue.

Issue (year)	Author/manuscript	# Citations (JACMP)	# Citations (Google Scholar)
6 (2005)	Renner WD, et. al., A method for deconvolution of integrated electronic portal images to obtain incident fluence for dose reconstruction, 2005.	44	127
7 (2006)	Wink NM, et. al., Phase versus amplitude sorting of 4D‐CT data, 2006.	67	202
8 (2007)	Yoon M, et. al., A new homogeneity index based on statistical analysis of the dose–volume histogram, 2007.	94	188
9 (2008)	Yap MH, et. al., A novel algorithm for initial lesion detection in ultrasound breast images, 2008.	65	122
10 (2009)	Yan G, et al., On the sensitivity of patient‐specific IMRT QA to MLC positioning errors, 2009.	89	154

*Note*: The system currently used by Wiley underestimates JACMP manuscript citations relative to Google scholar. Citation numbers are current as of 1/5/2024.

In addition to leading the effort to identify a new publisher, Michael Mills worked tirelessly on a number of issues, including the important task of getting the JACMP included in the Thomson ISI (Institute for Scientific Information) citation databases. This allowed JACMP manuscripts to be indexed and searched, and for authors and others to identify the number of article citations and their referencing sources. This also allowed the JACMP to receive its first journal impact factor; the most recent (2022) journal impact factor for the JACMP is 2.1. For comparison, those for *Medical Physics* and *Physics in Medicine and Biology* are 3.8 and 3.5, respectively (source: Wiley communication, 22/4/2024). The JACMP was first abstracted and indexed in 2006 in issue 6.1.

In September, 2006, again thanks to Michaels's efforts as well as those of Azam Niroomand‐Rad, the International Organization of Medical Physics (IOMP) agreed to co‐sponsor the JACMP. This immediately increased the global visibility of the Journal, and the number of submitted manuscripts quickly doubled (though this did not immediately lead to a commensurate increase in the number of published manuscripts). In response, the JACMP recruited three additional associate editors and asked IOMP to recommend four more. Most significantly, this put the JACMP on a path that would become the global resource for clinical medical physics. Key aspects of the agreement included:

- The JACMP web site would reference the IOMP as a “co‐sponsoring” organization.
- The IOMP web site would note that the JACMP was co‐sponsored by IOMP.
- JACMP articles would be free to download for anyone with web access worldwide.
- Authors of JACMP articles retain the copyright and may post or publish their articles anywhere for unlimited distribution as long as the JACMP is credited as the publication of record.

In an effort to encourage younger physicists to join at an earlier stage in their career, the ACMP added a new membership classification of “In‐Training” in 2006. Along with this, a Young Investigator's Symposium was added to the annual meeting beginning in 2007, and accepted abstracts were published in the JACMP (in issues 8.3, 9.3, and 10.3).

In 2006, Michael informed the ACMP that he would be stepping down as JACMP editor‐in‐chief at the end of 2007. At the time, I was the ACMP chairman, and Herb Mower (ACMP Vice‐Chair) and I formed an ad‐hoc committee to search for Michael's replacement. Qualifications outlined by the committee included:

- The individual selected should have held office or served on the Board of either the American Association of Physicists in Medicine or the American College of Medical Physics.
- The individual selected should have at least 10 years of experience in publishing scientific, clinical and professional articles. Experience may include a previous tenure as Editor‐in‐Chief or Associate Editor of Medical Physics, Physics in Medicine and Biology or the International Journal of Radiation Oncology, Biology and Physics.

Additional desirable experience included:

- Educational experience, including the direction of a CAMPEP accredited training or residency program in Medical Physics is desirable.
- Research experience, including the direction of a research program at a major institution.
- Administrative experience, including the direction of a medical physics clinical support program.
- National honors, such as AAPM and/or ACMP Fellow, or the William D. Coolidge award from the AAPM or the Marvin M. D. Williams award from the ACMP.

I note with some amusement that the recruitment document also stated the estimated time involvement for the incoming editor‐in‐chief was “….5 h per week or 20 h per month.” That was an understatement at the time, and not remotely close to the effort required to publish the JACMP now.

On 1 January 2008 beginning with issue 9.1, George Starkschall assumed the role of JACMP editor‐in‐chief. George opened his tenure with an editorial on impact factor (at that time, the JACMP was still awaiting its first impact factor). George astutely recognized that there is more to the value of a journal than just an impact factor: “The real measure of impact of a paper in the Journal of Applied Clinical Medical Physics, then, is the number of times the information in that paper is used by a practitioner of medical physics to improve the quality of medical physics practice.” This philosophy continues to the present day, and notably, JACMP manuscripts were downloaded 1.17 million times in 2023 (source: Wiley communication, 22/4/2024).

In closing, I would like to highlight two other editorials published during this period. The first was by JACMP editor‐in‐chief George Starkschall in issue 9.2 in June, 2008.^1^ The editorial was concurrent with recent decisions within the AAPM and ABR regarding education and training requirements for board certification.* There was a growing concern (subsequently well justified) that these requirements could lead, in part, to at least a short‐term shortage of medical physicists. To ameliorate such a shortage, the concept of a new educational model, a “professional doctorate degree” in medical physics (often referred to as a doctorate in medical physics, or DMP degree) was in the early stages of conversation. George provided an excellent summary of the pros, cons, and potential consequences of such a degree, and it is interesting now to reflect back on those. Ultimately, the professional doctorate degree in medical physics has had little impact on the workforce challenges, as to date, only four programs have been established and accredited by CAMPEP.

The second editorial is a guest editorial from founding editor Peter Almond entitled *Grimmett and the Cobalt Unit*.^2^ Leonard George Grimmett was a British physicist, and early in his career worked at the London Radium Institute and at Westminster Hospital in London. Beginning in the 1930s and before he had completed an advanced degree, Grimmett made many notable contributions to the field we now call medical physics. In addition to the design of early megavoltage teletherapy devices described by Peter,^2^, ^3^, ^4^ Grimmett made contributions to nuclear medicine and radiation biology.^5^, ^6^ Of particular note, in 1935 Grimmett described the production of radioactivity by neutrons^7^ (recall that the neutron had only been discovered 3 years earlier by Sir James Chadwick). Grimmett was subsequently recruited to MD Anderson in 1949 (the same year as Gilbert Fletcher was recruited) specifically to build the first Co‐60 unit^8^, ^9^; sadly, Grimmett died unexpectedly before it could be completed. My point here is not to recap Grimmett's life and distinguished career; for that I refer the interested reader to a book Peter published in 2013 entitled *Cobalt Blues: The Story of Leonard Grimmett, the Man Behind the First Cobalt‐60 Unit in the United States*.^10^ Rather, I wanted to note that many of Grimmett's publications were in the journal *Nature*, a journal with an impact factor of 64.8 (2002) and largely beyond the reach of medical physics research today.

## Footnotes

* Recall that beginning in 2012, in order to take the ABR Part 1 Examination in Radiologic Physics, candidates must have been enrolled in or have graduated from a CAMPEP accredited education program (MS, PhD, residency). And beginning in 2014, in order to take the ABR Part 1 Examination in Radiologic Physics, candidates must have been enrolled in or have completed a CAMPEP accredited medical physics residency.
